# An incremental clustering method based on the boundary profile

**DOI:** 10.1371/journal.pone.0196108

**Published:** 2018-04-20

**Authors:** Junpeng Bao, Wenqing Wang, Tianshe Yang, Guan Wu

**Affiliations:** 1 Department of Computer Science & Technology, Xi’an Jiaotong University, Xi’an, P.R. China; 2 China Xi’an Satellite Control Center, Xi’an, P.R. China; Southwest University, CHINA

## Abstract

Many important applications continuously generate data, such as financial transaction administration, satellite monitoring, network flow monitoring, and web information processing. The data mining results are always evolving with the newly generated data. Obviously, for the clustering task, it is better to incrementally update the new clustering results based on the old data rather than to recluster all of the data from scratch. The incremental clustering approach is an essential way to solve the problem of clustering with growing Big Data. This paper proposes a boundary-profile-based incremental clustering (BPIC) method to find arbitrarily shaped clusters with dynamically growing datasets. This method represents the existing clustering results with a collection of boundary profiles and discards the inner points of clusters rather than keep all data. It greatly saves both time and space storage costs. To identify the boundary profile, this paper presents a boundary-vector-based boundary point detection (BV-BPD) algorithm that summarizes the structure of the existing clusters. The BPIC method processes each new point in an online fashion and updates the clustering results in a batch mode. When a new point arrives, the BPIC method either immediately labels it or temporarily puts it into a bucket according to the relationship between the new data and the boundary profiles. A bucket is employed to distinguish the noise from the potential seeds of new clusters and alleviate the effects of data order. When the bucket is full, the BPIC method will cluster the data within it and update the clustering results. Thus, the BPIC method is insensitive to noise and the order of new data, which is critical for the robustness of the incremental clustering process. In the experiments, the performance of the boundary point detection algorithm BV-BPD is compared with the state-of-the-art method. The results show that the BV-BPD is better than the state-of-the-art method. Additionally, the performance of BPIC and other two incremental clustering methods are investigated in terms of clustering quality, time and space efficiency. The experimental results indicate that the BPIC method is able to get a qualified clustering result on a large dataset with higher time and space efficiency.

## Introduction

One of the most important features of Big Data is that the collection of data is quickly and continuously expanding despite the huge amount of data. The applications include satellite monitoring, financial transaction administration, web information processing and more. It is straightforward but untenable to cluster all data from scratch every time any new data arrive. The incremental clustering approach is a way to address the dynamically growing dataset. It attempts to minimize the scanning and calculation effort for newly added data points[1]. It is essential to efficiently store and utilize knowledge about the existing clustering results for incremental clustering.

This paper proposes a boundary-profile-based incremental clustering (BPIC) method, which represents the clustering results using a collection of boundaries while all inner data of clusters are ignored. A boundary-vector-based boundary point detection (BV-BPD) algorithm is also proposed to capture the boundary profiles. The boundary profile is helpful to record knowledge with less memory. In addition, it provides an easy way to label the new data and update the clustering results. When a new data point arrives, the BPIC first identifies the relationship between it and the boundary profiles. If it belongs to a boundary profile, the new point will be labelled accordingly. Otherwise, it is temporarily preserved in a bucket since it could be either noise or a seed of a new cluster. When the bucket is full, a DBSCAN[2] (Density-Based Spatial Clustering of Applications with Noise) clustering algorithm, which is robust to noise, is employed to cluster the data within it. At last, the BPIC method merges the overlapping cluster boundaries. The bucket can help to not only distinguish the noise from the seeds of new clusters but also alleviate the effect of data point ordering.

There are many studies[3,4,5,6] about stream clustering, which also addresses the continuously changing dataset. However, it is different from incremental clustering. Most stream clustering methods (such as[7,8,9,10]) make the basic assumption that users are more interested in the new data rather than the old. Therefore, the historical data are forgotten as data streams evolve. However, the BPIC method does not forget any old data because it is believed that the old information is as important as the current or new data. This is a common situation in many applications, such as the fraud detection, health care and others. For any stream clustering method, it desires a data structure to store statistical features of data streams in memory[11], such as corset trees[7], CF vector[8], and grids[10]. However, it is difficult to design a set of universal features for different data of varying natures. Thus, the BPIC method operates on the raw data instead of extracting features.

The main contributions of this paper are as follows. (1) A new boundary point detection method BV-BPD is proposed, which outperforms the state-of-the-art method. (2) An incremental clustering method BPIC is presented. It exploits boundary profiles to represent knowledge instead of using all data points, which greatly improves time and space efficiency. (3) The BPIC method can deal with noise and find arbitrarily shaped clusters. (4) The BPIC method is insensitive to the order of data points, which is critical for the robustness of the incremental clustering process.

## Related work

### Incremental clustering method

Clustering is an unsupervised way to divide the dataset into several groups so that data points are similar within a group and different between groups. Clustering methods have been studied for many decades. They can be divided into five categories, including hierarchy based, partition based, density based, grid based and model based clustering. Each category has its characteristics. For hierarchical clustering, once a decision is made to combine two clusters, it cannot be undone. For partition based clustering, the number of clusters has to be specified prior to the process. Both of them are sensitive to noise and outliers and prone to globular-shaped clusters. However, for the density based clustering method, it can discover clusters of arbitrary shapes and handle the noise. Grid based clustering methods divide the object space into a finite number of grids or cells and then perform clustering operation on the grids that are not empty. The biggest advantage of grid based methods is the low computation complexity for high dimensional data since it depends on the number of grids in each dimension rather than the amount of data. But it is non-trivial to determine the parameter of grids, which has the significant effects on the clustering results. The basic idea of model based methods is that the objects within one cluster are of the same distribution in statistics. However, it is not suitable for the dataset with a large number of clusters and a small number of objects. The specific introductions of the clustering methods are in [12,13]. Some of the clustering methods are of an incremental manner, such as BRICH and COBWEB. However, most of them cannot be directly applied to the growing datasets. Many incremental clustering methods are derived from the traditional clustering methods. Thus, from our viewpoint, incremental clustering methods are categorized into three main groups: density based, hierarchy based and partition based.

Ester et al.[14] first proposed the density based incremental clustering algorithm, which is based on DBSCAN, for mining in a data warehousing environment. Due to the dense of DBSCAN, the insertion and deletion of a new point affects only its neighbourhood. This incremental DBSCAN can yield the same result as performing the DBSCAN from scratch when new data arrive, but the clustering is inefficient since updates are processed one at a time without considering the relationships between the single updates.

Chen et al.[15] introduced the hierarchical based incremental clustering algorithm GRIN, which is based on gravity theory. There are two phases in GRIN. In the first phase, the GRIN builds a clustering dendrogram for a number of samples buffered in the pool and then flattens and prunes the bottom levels of the dendrogram in order to derive the tentative dendrogram. In the second phase, the GRIN determines whether each data in the pool should be inserted into the leaf clusters in the tentative dendrogram. If the data belongs to more than two leaf clusters, the principle of gravity is employed to determine its ultimate leaf cluster. Although GRIN has linear time complexity and is insensitive to the data input order and the parameters, it is not really an incremental clustering algorithm but is a batch mode method since all new data are first buffered in an incoming data pool.

Patra et al.[16] proposed a distance based incremental clustering method al-SL that can find arbitrarily shaped clusters, which is indeed a partitional clustering method. It determines the membership of each new data point according to the distance between the data point and the corresponding closest leader, and detects whether there exists an affected region. However, it is time consuming to scan all leader points and identify the key leaders of each new data point in a large dataset. In addition, the incremental al-SL method is sensitive to noise.

Bandyopadhyay and Murty[17] proposed an axiomatic framework for incremental clustering that considers quality and computational complexity. They presented an FP Tree based incremental clustering algorithm. However, the incremental frequent pattern tree can deal with only discrete or categorized data rather than continuous real value data. Ackerman and Dasgupta[18] proved that the memory-bound incremental method is weaker than the batch mode in terms of cluster structure detection. However, it is difficult to make a distinction between noise and a seed of a new cluster when processing one data point at a time.

There are also some other studies about incremental overlapping clustering[19,20] in which a data point can belong to several clusters. Nonetheless, it is out of the scope of this paper. In this work, a density based incremental clustering BPIC is proposed. In many real-time applications, the goal is to identify the new point and discover its cluster as soon as it arrives and updates the clustering results when the structures change. Frequent updates of clustering results is unnecessary since some new data points will not change the current clustering results. Moreover, frequent updates will reduce the time efficiency. Therefore, in the BPIC, each new point is processed in an online fashion and the clustering results are updated in a batch mode. The clustering results are represented by the boundary profiles, which is the foundation of the BPIC. The studies about boundary point detection are as follows.

### Boundary point detection

Ester et al.[2] introduced the density clustering method DBSCAN and proposed the concept of boundary points. However, there is no discussion about how to efficiently detect boundary points. Qiu and his team[21–25] have been studying the Boundary Points Detection (BPD) problem for many years. Qiu et al.[21] proposed a typical density-based BPD algorithm called BRIM. It defines the boundary point as the one whose boundary degree is greater than a given threshold δ. However, it is difficult to estimate the optimal threshold δ. They also come up with a BPD algorithm based on grid entropy[22], which measures the distribution uniformity of data points within a cluster. It is supposed that the boundary points should fall into the grids with small entropy. However, this method requires more parameters, including the size of the grids, a density threshold and an entropy threshold. Later, they presented a clustering BPD algorithm based on gradient binarization[23], which adopts image edge detection. This algorithm uses grids to enhance the speed and the Prewitt gradient operator to calculate the grid gradient. The larger the gradient is, the more likely it is the boundary. The objects within boundary grids are boundary points.

Xia et al.[26] presented a BPD method, BORDER, based on the reverse k-nearest neighbour. They believe that boundary points (as well as noise/outliers) have fewer reverse k-nearest neighbours than data points within clusters. However, BORDER cannot distinguish noise from boundary points. In addition, a user must decide the number of boundary points, which makes it difficult to use. Zhang et al.[27] proposed a DDBound method to cluster and detect the boundary of streaming data. Recently, Qiu et al.[28] introduced a clustering boundary detection method by the transformation of affine space (called BD-AFF) that outperforms BRIM and BORDER according to the experimental results. Tong et al. [29] argued that boundary points are essential for clustering because they represent the distribution of the dataset. Therefore Tong et al. proposed the Scalable Clustering Using Boundary Information (SCUBI), which could reduce the running time. The basic idea of SCUBI is that the clustering process is performed only on the selected boundary points rather than the entire dataset. And the rest data points are then assigned to the same cluster as their nearest boundary points.

## The BV-BPD algorithm

For incremental clustering, it is essential to utilize the knowledge about existing clusters to label the new data and update the whole clustering results. To record the knowledge with less memory, the boundary profile is used to represent a cluster in this paper. Thus, boundary profile detection is the basis of the proposed BPIC method, which is utilized to identify the new data point and update the whole clustering results in the BPIC. This paper proposes the concept of boundary vector (BV) and the BV-based boundary point detection (BV-BPD) algorithm to capture the boundary profiles.

Although there are many BPD methods in the literature, each of them still has some problem. For example, BORDER cannot deal with noise. BRIM is not suitable for the high dimensional data. BORDER, BD-AFF, and SCUBI require the parameter of the boundary points proportion. So this paper presents the BV-BPD algorithm to address these issues. Comparing with other BPD methods, the BV-BPD algorithm is robust to noise and can automatically distinguish the boundary points and internal points without a given threshold. In addition, it can be easily extended to high dimensional data.

### Boundary vector

**Fig 1:**
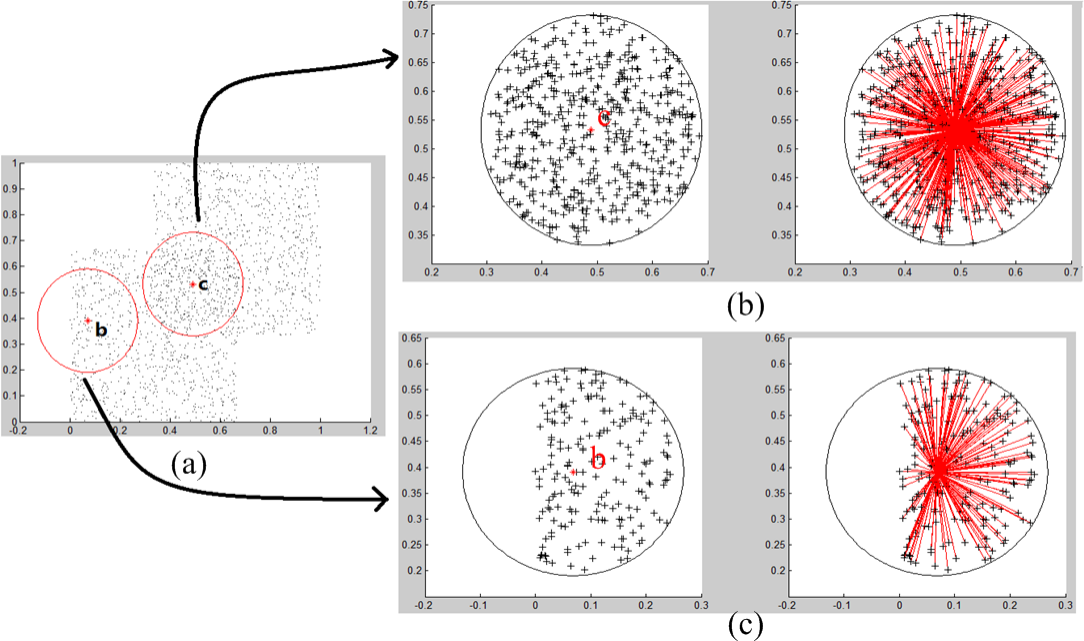
The density distribution of a core point *c* and a boundary point *b*. (a) Point b and c are boundary and core point respectively. (b) The boundary vector of core point c within its neighbourhood. (c) The boundary vector of boundary point b within its neighbourhood.

Typically, a cluster can be represented by its centroid vector, which is suitable for the spherical shaped cluster. To characterize any shaped clusters, this paper exploits a density-based boundary point detection approach. Usually, boundary points are located at the margin of a cluster where there is a density cliff. In most cases, clusters are separated or surrounded by sparse regions. Naturally, there is a boundary between a dense region and a sparse region. A core point is in the dense region inside a cluster, while an isolated point or noise is in the sparse region. A point located at the border is referred to a boundary point. Thus, there is a density cliff in a boundary point’s neighbourhood. Fig 1 illustrates the density distribution of a core point *c* and a boundary point *b*. Therefore, if a density unbalance is detected in a point’s neighbourhood, then the point is a boundary point.

Definition 1 Candidate Core Point. A candidate core point is a data point *c* in data set D that satisfies the following condition.

$$\left\{\forall c|c\in D,\rho_{r}(c)>\tau \right\}$$ (1)

where *ρ_r_*(*c*) denotes the density of point *c* in its neighbourhood of radius *r*. *τ* denotes a density threshold. In fact, the value of *ρ_r_*(*c*) can be calculated by the number of points in the neighbourhood within radius *r*. If *ρ_r_*(*c*) < *τ*, point c is the noise.

The above definition suggests that a point whose neighbourhood is of high density is a candidate core point. In Fig 1A, point b is a boundary point, and c is a core point. Their neighbourhoods are marked by red circles, and the corresponding neighbourhood densities are shown in the left part of Fig 1B and Fig 1C. For the boundary point b, one side of the neighbourhood is dense and the other side is sparse. However, the density over the entire neighbourhood may exceed the threshold τ. This example indicates that for a boundary point, its neighbourhood density may still be greater than τ. Thus, a core point cannot be distinguished from a boundary point just by the neighbourhood density. This is why it is called a candidate core point.

To differentiate the boundary point from the candidate core point, the notion of the boundary vector is introduced, which is defined as follows.

Definition 2 Boundary Vector. The boundary vector of a point p is the sum of all directed vectors originating from p and it satisfies the following condition.

$$\vec{P}=\frac{\sum_{q_{i}\in \rho_{r}(p)}\vec{pq_{i}}}{\left|\rho_{r}(p)\right|}$$ (2)

where $\vec{P}$ denotes the boundary vector of a point p. *ρ_r_*(*p*) denotes the neighbourhood density of point p with radius *r*. $\vec{pq_{i}}$ denotes a directed vector from point p to point q_i_, which is in p’s neighbourhood. Each red line in Fig 1B and Fig 1C represents a vector $\vec{pq_{i}}$.

The boundary vector of point p has two features.

1. The norm of the boundary vector tends to be 0 if the points in p’s neighbourhood are of uniform distribution, which implies that p is a core point. Since all vectors have the same starting point p and the end point may be distributed in any direction. It is very likely that each vector has an opposite vector in the neighbourhood. Fig 1B illustrates this situation.
2. The norm of a boundary vector deviates from 0 if the distribution of points in p’s neighbourhood is non-uniform, which implies that p is a boundary point. Fig 1C illustrates this situation. Therefore, the norm of the boundary vector can be used to distinguish a boundary point from a core point.

Additionally, for a boundary point p, one side of its neighbourhood is dense, while the other side is sparse. It can be concluded that the boundary vector of p is always directed towards the dense region because most $\vec{pq_{i}}$ end in the dense region and have fewer opposite vectors in the sparse region. Thus, the final boundary vector of p will point towards the dense area.

Definition 3 Core Point. A core point is a data point *c* in the data set D that satisfies the following condition.

$$\left\{\forall c|c\in D,\rho_{r}(c)>\tau ,|\vec{C}|<\lambda \right\}$$ (3)

where $\left|\vec{C}\right|$ is the norm of point *c*’s boundary vector. λ is a boundary threshold that can be automatically obtained by a k-means clustering procedure rather than being manually set. The above definition suggests that if a point is a candidate core point and its boundary vector norm is small enough, then it is a core point.

This heuristic rule can divide the candidate core points into two classes: boundary points and core points. Indeed, a bisecting k-means method (k = 2) is adopted to obtain two clusters after the low-density points are filtered and the noises are removed. A cluster that contains boundary vectors with larger norms is a collection of boundary points. Otherwise, it is a set of core points.

**Fig 2:**
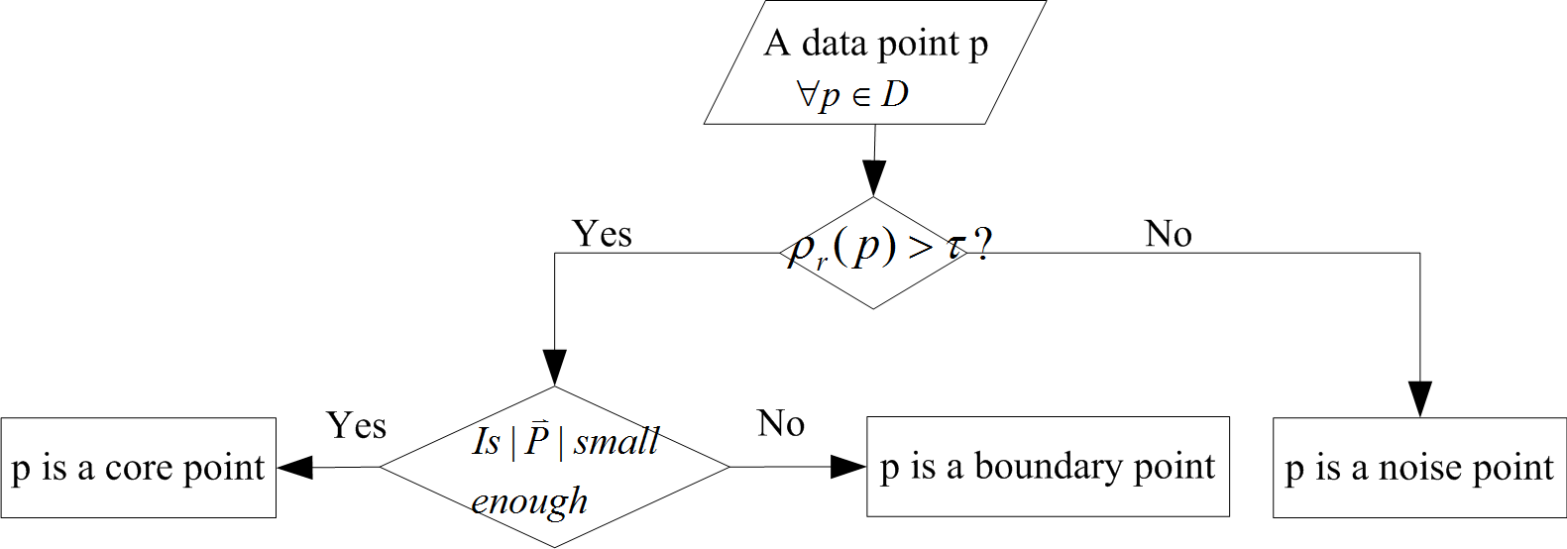
The discriminant tree of a data point.

According to the above definitions and facts, Fig 2 summarizes the discriminant tree for a data point.

### Boundary point detection algorithm

Based on the Boundary Vector concept, a boundary-vector-based boundary point detection (BV-BPD) algorithm implements the discriminant tree mentioned above. The algorithm first removes the noise and then exploits a bisecting k-means algorithm to partition all points into two clusters. One cluster is a set of boundary points, and the other is a set of core points. BV-BPD is an adaptive algorithm that automatically separates core points from boundary points according to the differences between the boundary vectors’ norms rather than according to a user-defined threshold. The pseudocode of BV-BPD is as follows.

*Input*: *a data set D*, *neighbourhood radius r*, *density threshold τ*.

*Output*: *a set of boundary points*.

Begin

Step1: calculate the neighbourhood density and boundary vector of each point in D by Eq 2;

*Step2*: *remove noise if the point neighbourhood density is lower than τ*;

*Step3*: *execute bisecting k-means clustering method on the boundary vector’s norm of all points;*

*Step4*: *the cluster with larger boundary vector norm is the set of boundary points*, *denoted as bp*, *and the other cluster is a set of core points*, *denoted as cp;*

*Step5*: *return the boundary point set bp*.

End

## The BPIC method

### The basic ideas

There are two basic problems in the incremental clustering. The first is how to quickly identify the cluster of the new incoming data. The second is how to update the clustering results when the structures change. With new data continuously arriving, new clusters will emerge and some clusters may be connected. It should be noted that no cluster will disappear since the old data are not forgotten in our assumption. In many applications, the former is required to be processed in real time, while the latter is desired to be executed when the clustering results change. Frequent updates are unnecessary and unfavourable since many new data points just fall into the inside of the existing clusters, which will not change the clustering results. In addition, frequent updates will have negative effects on time efficiency. Thus, the BPIC method processes each new data in an online fashion and updates the clustering results in a batch mode.

First, the DBSCAN algorithm is used to cluster all existing data points and obtain the initial clusters. Then, the proposed BV-BPD algorithm is used to obtain the boundary profile of each cluster. The BPIC method exploits the last clustering results, which are represented by the boundary profiles, to process new data. If a new point belongs to existing clusters, it will be immediately labelled the corresponding cluster. Otherwise, it is temporarily preserved by a bucket due to the uncertainty regarding whether it is noise or a seed of a new cluster. As these uncertain data accumulate, new clusters might emerge, and some clusters may be connected or combined. When the bucket is full, the BPIC method updates the entire clustering results.

In the following section, the paper focuses on two operations, i.e., identifying new incoming data based on existing boundary profiles and updating the clustering results when the bucket is full.

### Identifying the new points

There are three kinds of relationships between a point *p* and a cluster’s boundary profile *B*.

1. If the distance between *p* and a boundary point *b* that belongs to *B* is less than the neighbourhood radius, then *p* is on the boundary profile, which is denoted as *p*⊥*B*.
2. If there is a boundary point *b* that belongs to *B*, and the distance from *p* to the end of *b*’s boundary vector is less than the distance from *p* to the beginning of *b*’s boundary vector, then *p* is inside of the boundary profile, which is denoted as *p*⊕*B*.
3. If it does not satisfy the above two conditions, the point *p* is outside of the boundary profile, which is denoted as *p*#*B*.

Eq (4) formally defines these three relationships.

$$\left\{ \begin{array}{l} p⊥B≔\{\exists b|b\in B,\;dist(p,b)\leq r\}\\ p⊕B≔\{\exists b|b\in B,\;dist(p,b_{end})<dist(p,b)\}\\ p\#B\;otherwise \end{array}\right.$$ (4)

where *b* is a boundary point, *B* is the boundary profile of a cluster, *dist*(*p*,*b*) denotes the distance between *p* and *b*, *r* is the neighbourhood radius, and *b*_*end*_ denotes the end of *b*’s boundary vector.

**Fig 3:**
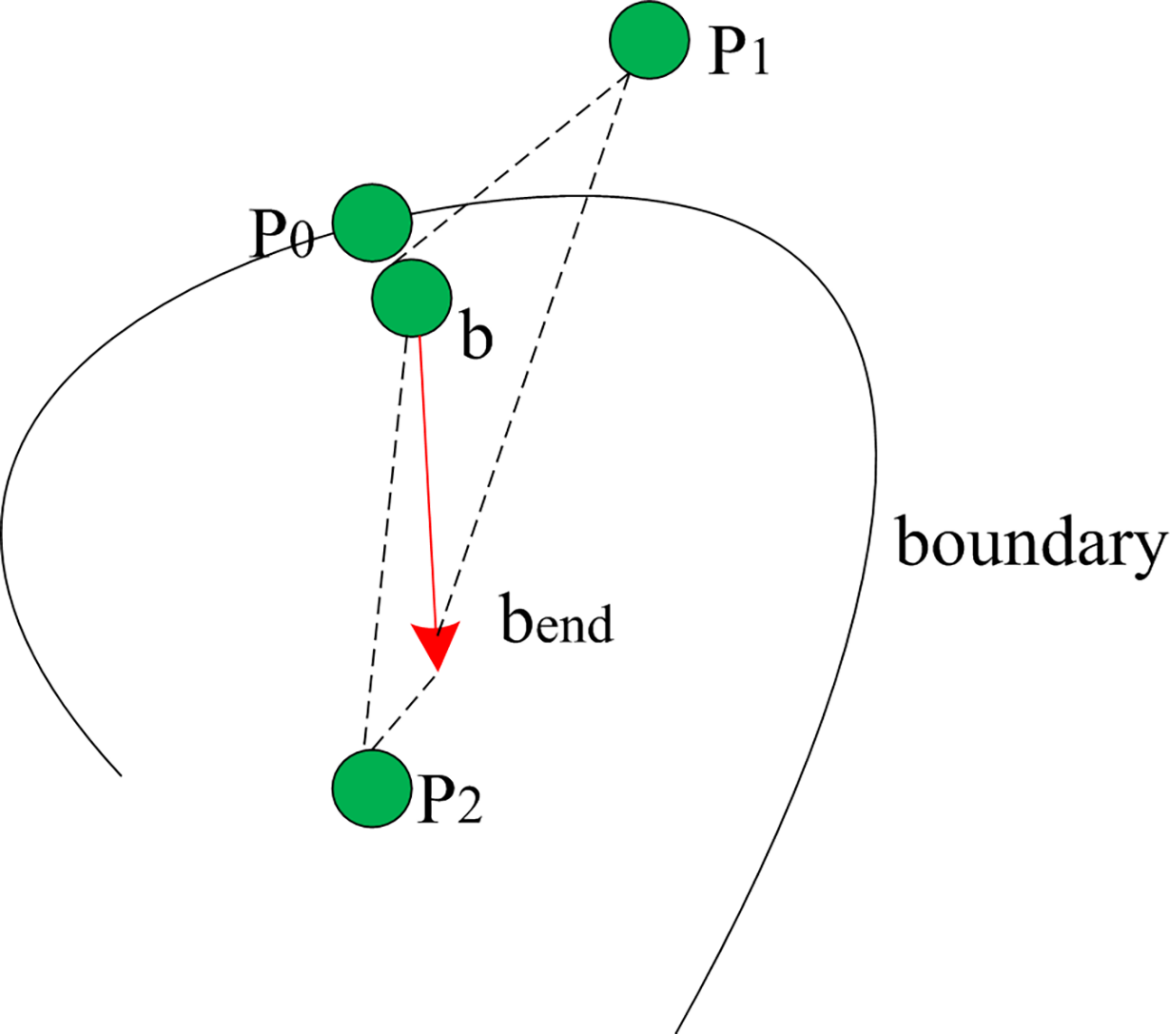
The relationship between a point and a boundary vector. *b* is a boundary point and its boundary vector is marked with a red arrow. *b*_*end*_ represents the end of *b*’s boundary vector. *P*_*0*_ is a point in the neighbourhood of *b* and it becomes a new boundary point. *P*_*1*_ is a point outside of the boundary profile since it is closer to *b* than to *b*_*end*_. *P*_*2*_ is a point inside of the boundary profile since it is closer to *b*_*end*_ than *b*.

The intuitive interpretation of this definition is that if a new point *p* is very close to a cluster’s boundary point, then *p* could be absorbed into this boundary profile. When *p* is inside of the boundary profile, it should be closer to the end of a boundary vector than to the start since the boundary vector is always directed towards the dense region inside the cluster. Fig 3 illustrates these situations.

For a new point that is inside of a cluster, only the corresponding cluster label will be returned to the user. It will not be maintained in the memory since the internal points will not contribute to the change of clustering results. For a new point that is a boundary point or outside of any cluster, it will be kept in the memory. The pseudocode of identifying a new incoming data point by BPIC is as follows.

*Input*: *a new data point p*, *a list of boundary profiles BP*, *grid size d*, *neighbourhood radius r*, *bucket*.

*Output*: *the label of p*.

Begin

*Step 1*: *divide data space into grids*. *grid*_*p*_*(x*,*y) denotes that p falls into grid(x*,*y); x and y indicate the grid location*.

*Step 2*: *obtain p’s list of neighbouring points*, *denoted as plist*, *which is the set of points in grid*_*p*_ *(x-1*,*y-1)*, *grid*_*p*_*(x-1*,*y)*, *grid*_*p*_*(x-1*,*y+1)*, *grid*_*p*_*(x*,*y-1)*, *grid*_*p*_ *(x*,*y)*, *grid*_*p*_ *(x*,*y+1)*, *grid*_*p*_ *(x+1*,*y-1)*, *grid*_*p*_ *(x+1*,*y) and grid*_*p*_ *(x+1*,*y+1)*.

*Step 3*: *calculate dist(p*, *neighbourb*_*i*_*)*, *neighbourb*_*i*_∈*plist*.

*Step 4*: *find a point b that minimizes dist(p*,*b) where b*∈*plist and b*∈*boundary profile S*_*i*_.

*Step 5*: *if dist(p*,*b)<r*, *then p is on the boundary S*_*i*_, *which corresponds to cluster C*_*i*_*; then add p into boundary profile S*_*i*_ *and put it into the bucket*. *Return C*_*i*_.

*Step 6*: *if dist(p*,*b*_*end*_*) <dist(p*,*b)*, *then p is inside of the boundary S*_*i*_ *and p is a new member of the cluster C*_*i*_. *Return C*_*i*_.

*Step 7*: *else p is outside of the boundary*, *and put p into the bucket*. *Return NULL*.

End

For the new data point *p*, in order to efficiently find its nearest boundary point *b*, first the data space is split into grids (step 1). The grid size *d* should be set greater than the neighbourhood radius *r*, which guarantees that *p’s* nearest point within the neighbourhood is located in the adjacent grids of *grid*_*p*_*(x*,*y)*. Therefore, only the nine adjacent grids of *grid*_*p*_*(x*,*y)* need to be scanned (step 2).

For a data point outside of any boundary profile, it is unreasonable to immediately treat it as noise since it might be a seed of a new cluster and a member of a potential cluster. With the accumulation of uncertain new data points, a new cluster may emerge. Thus, such uncertain data points are preserved in a bucket. When the bucket is full or the clustering results require updating, the BPIC method clusters the data within the bucket and then updates the entire clustering results. At this time, the data points in the bucket could be labelled. The update operation is discussed in the next section.

### Updating the clustering results

There are two modes of updating clustering results. One is the real-time mode in which clusters are updated when every new point arrives. Obviously, it wastes time since some points will not change the structure of the clustering results. The BPIC algorithm employs a batch mode update strategy. It uses a bucket to preserve the data points that are outside of any existing clusters, which could be either noise or seeds of new clusters. All of the clustering results are updated when the bucket is full. The pseudocode of the update process is as follows.

*Input*: *bucket*, *a set of existing boundary profiles BP*.

*Output*: *the updated boundary profile set*.

Begin

*Step 1*: *cluster the points within bucket by DBSCAN;*

*Step 2*: *obtain a set of boundary points Db in bucket by BV-BPD;*

*Step 3*: *obtain the boundary profile of each cluster*, *i*.*e*., *a list of the boundary profiles of Db*, *denoted by Dbp;*

*Step 4*: *for each Dbp*_*i*_ *in Dbp*:

*Step 5*:     *flag = 0*

*Step 6*:     *for each point in Dbp*_*i*_:

*Step 7*:         *if (point ∈ BP*_*j*_ *and BP*_*j*_ *∈BP)*:

*Step 8*:             *flag = 1*

*Step 9*:             *merge Dbp*_*i*_ *and BP*_*j*_

*Step 10*: *if (flag = = 0)*:

*Step 11*:         *Dbp*_*i*_ *is added into BP*

*Step 12*: *return the updated BP*.

End

In the first step, a DBSCAN algorithm is employed to cluster data in the bucket, which could remove the noise. Thus, BPIC can distinguish the noise from the seeds of new clusters. The BV-BPD is used to obtain the boundary profile of each new cluster by step 3. In steps 4–11, the new clusters and old clusters are updated according to their relationships. There are three kinds of relationships between a new cluster and the existing clusters. The corresponding update operations are as follows.

1. The new cluster is an isolated one if there is no boundary point shared with other clusters. In this case, the new cluster is added into the whole clustering results, which corresponds to steps 10–11.
2. The new cluster is inside one of the existing clusters. In fact, this situation would never occur in our method since the points of the new cluster are from the bucket, which does not contain any point inside the existing clusters. This implies that the points of new clusters cannot be the internal points of any old cluster.
3. The new cluster is connected with some old clusters if there is at least one boundary point that concurrently belongs to some old cluster’s boundary profile. Then, these clusters are merged into one cluster, which corresponds to steps 7–9.

To speed up the update process, there is a trick in which the newly detected boundary points are also preserved into buckets, which corresponds to the step 5 in the pseudocode that identifies the new points. If a new point p is identified as a boundary point, it will be labelled accordingly. When clustering the points within the bucket in the update process, point p will also be classified into a new cluster. Thus, if point p concurrently belongs to two different clusters, these two clusters should be merged.

## Experimental results

The experimental environment is as follows.

Operating System: Windows 7.

CPU: Intel(R) Core(TM) i7-2600 CPU @ 3.40 GHz.

Memory: 32 GB

Disk: 1 TB

### Experiment I

This experiment tests the performance of the boundary profile detection method BV-BPD and compares it with the state-of-the-art method BD-AFF[28] in terms of precision, recall and the F1-measure. The dataset employed in this experiment is the Chameleon DS3[1], which is an arbitrarily-shaped 2D synthetic dataset that contains 10,000 points. The boundary points in the dataset are manually labelled.

**Fig 4:**
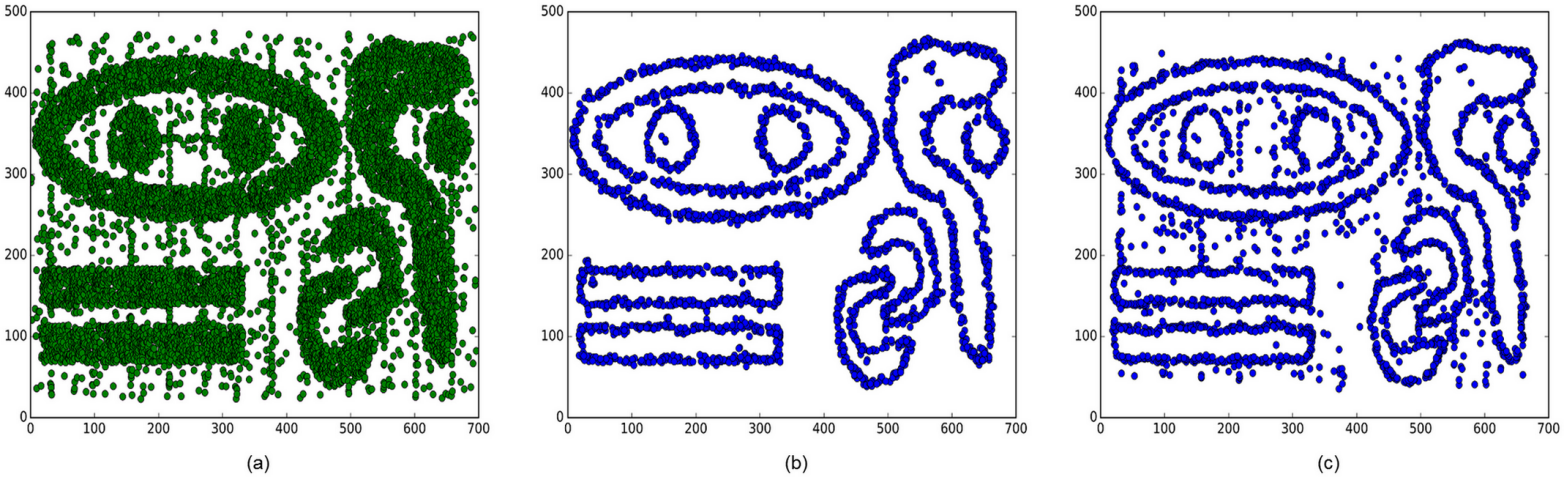
Chameleon dataset DS3 and the boundary points detection results of BV-BPD and BD-AFF methods. (a) The original data of Chameleon dataset DS3. (b) The boundary detection results of BV-BPD method. (c) The boundary detection results of BD-AFF method.

Our BV-BPD method has two parameters. The neighbourhood radius and the density threshold are set to 12 and 20, respectively, in this experiment. The BD-AFF method has three parameters. The neighbour quantity, the percentage of boundary points and the number of noise are set to 50, 0.3 and 800, respectively. Fig 4 shows the original Chameleon dataset DS3 and the boundary detection results of these two methods. It is obvious that the BV-BPD method is more robust to noise than the BD-AFF method. Table 1 compares the precision, recall and F1-measure evaluations of the two methods.

**Table 1:** Evaluation of the two methods’ boundary detection results.

method	precision	recall	F1-measure
BV-BPD	0.983	0.930	0.956
BD-AFF	0.765	0.740	0.752

### Experiment II

**Fig 5:**
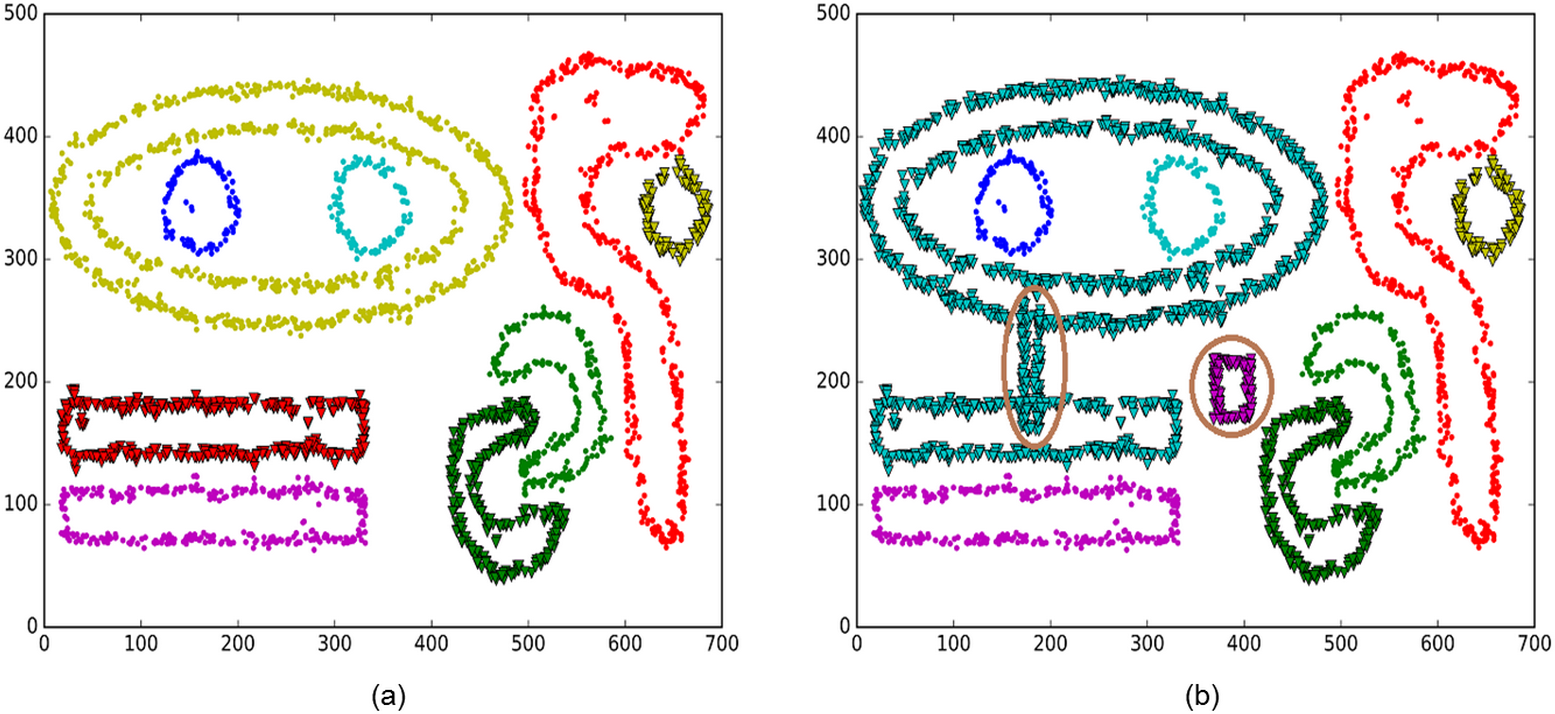
An incremental clustering results of the BPIC method. (a) The boundary profiles of the old clusters. (b) The final updated clustering results obtained by the BPIC method with 600 appended data points.

This section introduces two examples to illustrate the incremental clustering process of the BPIC method. In the first example, 600 new data points are generated and randomly added to the DS3 dataset, which is marked by two brown-coloured circles in Fig 5B. The old cluster boundary profiles are shown in Fig 5A. As a result, a new isolated cluster emerges as marked in the right circle. Two existing clusters are merged since they are connected by some new data points, which are shown in the left circle.

**Fig 6:**
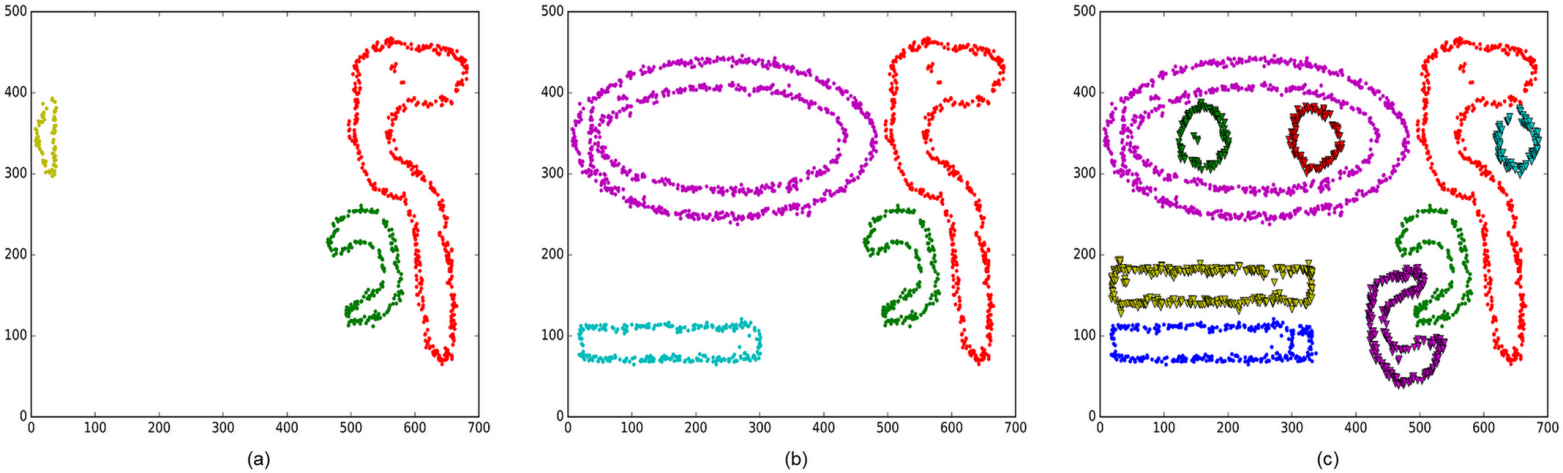
The evolution process of the BPIC results, in which the bucket size is 3500. (a) The initial boundary clustering results on 3000 data points from the Chameleon DS3 dataset. (b) The first updated clustering results after 3500 data points are added. (c) The second updated clustering results after 7000 data points are added.

Furthermore, Fig 6 shows the evolution process of the incremental clustering results. In the second example, the Chameleon DS3 dataset is split into two parts. One represents the old dataset (static dataset) that consists of 3000 data points, and the other represents the new incoming data that consists of 7000 data points. Fig 6A shows the initial clustering results of the old data. Fig 6B shows the first updated clustering results after 3500 new data points are added to the old dataset. Fig 6C shows the second updated clustering results after 7000 new data points arrive.

These two examples show that the BPIC method is able to properly cluster the growing dataset based on the existing results. Although the DS3 dataset contains noise, the BPIC method can distinguish the new cluster seeds from noise using the bucket strategy.

### Experiment III

This experiment evaluates the performance of the BPIC method in terms of clustering quality, time, and space efficiency on a large dataset. In addition, the BPIC is compared with the batch-mode DBSCAN and the distance based incremental clustering method al-SL[16].

In this experiment, the initial static data employed is the DS3 dataset. Then, 90,100 new data points are generated that contain 15,000 noise points. There are 100,100 total data points. The new data points arrive in a random order.

#### Clustering quality evaluation

**Fig 7:**
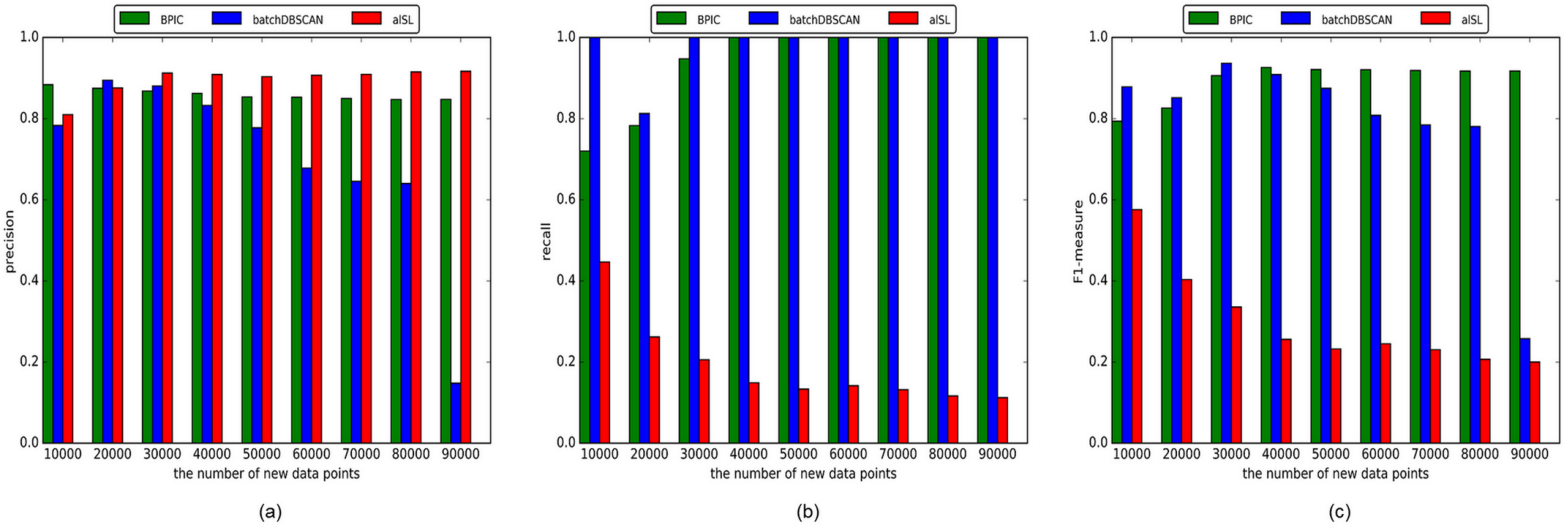
The evaluations of incremental clustering results by the three methods with different numbers of new points. (a)Precision of incremental clustering results. (b) Recall of incremental clustering results. (c) F1-measure of incremental clustering results.

For each method, the clustering results are evaluated in terms of precision, recall and the F1-measure when every 10,000 new points arrive. Fig 7 depicts the incremental clustering quality with the newly-added data points, varying from 10000 to 90000. It is shown in Fig 7A that both the BPIC and al-SL method have a high precision with different number of new points, while for the batch-mode DBSCAN the precision declines with as the number of new points increases. In Fig 7B, it is shown that the batch-mode DBSCAN method achieves the best recall with different numbers of new points. However, with more new points coming, the BPIC method has the same performance as the batch mode DBSCAN. For al-SL method, the recall is low and decreases as the number of new points increases. From Fig 7C it can be concluded that the BPIC outperforms the other two methods in terms of the F1-measure after the arrival of 40,000 new data points and the superiority is more evident with more data.

The batch-mode DBSCAN method cannot address the clusters with different densities. With new data, the distribution of the data density over the whole dataset may change, which leads to the lower precision. Similarly, the incremental al-SL method has a distance threshold that reflects the separation of clusters. The performance of the al-SL is getting worse if the distance threshold does not fit the density change with new data continuously coming. In addition, the al-SL method cannot address noise. For the BPIC method, it is not sensitive to the density change caused by the new incoming data since it keeps only the boundary points rather than the entire dataset. The density change of the boundary points will not be as significant as the inner points. This is another advantage of the boundary profile representation and the reason why the BPIC outperforms the other two methods when there is a large amount of new data.

**Fig 8:**
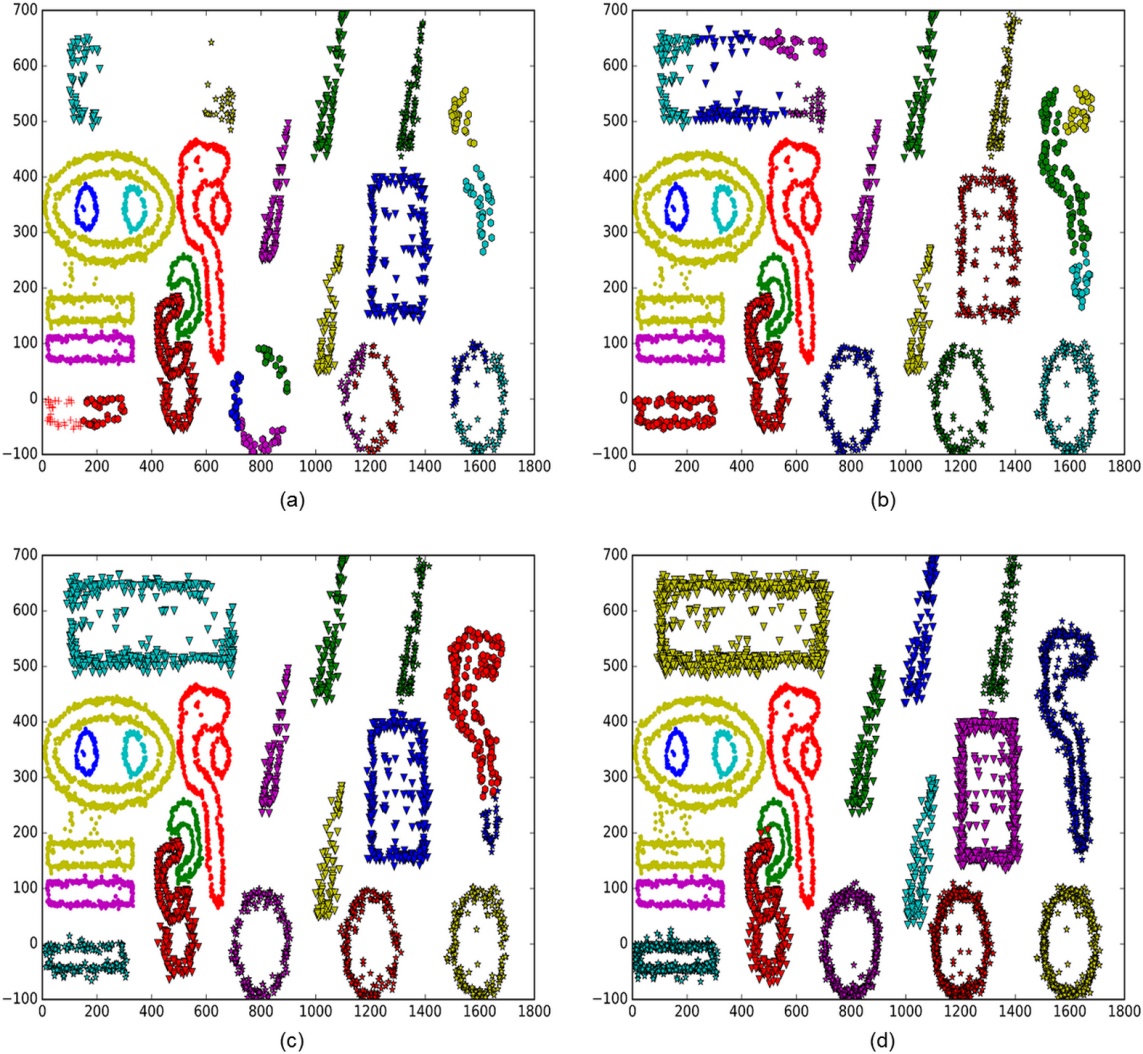
The incremental clustering results of the BPIC method with different numbers of new data points. (a) 10,000 new data points. (b) 20,000 new data points. (c) 30,000 new data points. (d) 90,000 new data points.

Fig 8 displays the incremental clustering results of the BPIC method with different numbers of new points. It can be seen that the boundary profiles gradually improve with more data.

#### Time and space efficiency

**Fig 9:**
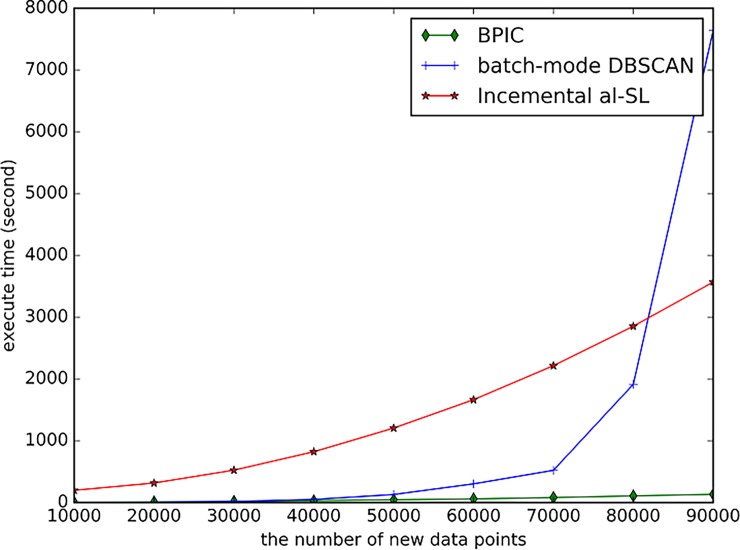
The execution time of the three methods against different numbers of new data points.

Fig 9 shows the execution time of the three methods with numbers of new data points that vary from 10,000 to 90,000. The bucket size of the BPIC method is 3,500. To have a fair comparison, the batch-mode DBSCAN method updates the clustering results from scratch every 3,500 new points, which is the same as the bucket size. As shown in the figure, for the BPIC method, the execution time slowly increases as new data points are added, and it is always shorter than the other two methods. For the batch mode DBSCAN, there is a sharp increase when the number of new data points achieves 80,000. The time complexity of DBSCAN is *O(n*^*2*^*)*, where n is the number of all data points. Thus, batch mode DBSCAN cannot deal with large scale data. The BPIC method is time efficient, although the DBCAN is also employed since the number of data to be clustered is small and fixed since *n* equals the bucket size. Thus, with new data continuously coming, the run time of the BPIC is shorter than the batch mode DBCSAN and this advantage is more obvious when the data is large.

In this experiment, the memory usage of these three methods with different numbers of new data points coming is investigated. Table 2 lists the storage space required by the three methods, which is measured by the number of data points stored in the memory. The original static dataset contains 10,000 data points and should be included in the incremental clustering process. The BPIC method retains only the boundary points of each cluster, while the other two methods retain all data points in memory during the whole process of incremental clustering. The storage saved by the BPIC is calculated as 1 minus the percentage difference between the number of boundary points and the number of all points. As shown in Table 2, the larger the number of new data points is, the more space is saved by the BPIC method. Thus, the BPIC method is suitable for the growing large data.

**Table 2:** The number of data points maintained in memory by each method with different numbers of new data points.

The number of new data points	BPIC	Batch-mode DBSCAN	al-SL	Percentage of storage saved by BPIC
10,000	4,315	20,000	20,000	78.42%
20,000	4,901	30,000	30,000	83.66%
30,000	5,460	40,000	40,000	86.35%
40,000	5,834	50,000	50,000	88.33%
50,000	6,321	60,000	60,000	89.46%
60,000	6,820	70,000	70,000	90.25%
70,000	7,314	80,000	80,000	90.85%
80,000	7,836	90,000	90,000	91.79%
90,000	8,156	100,000	100,000	91.84%

## Discussion

### Time complexity of the BPIC method

The process of the BPIC method includes three stages. First, when a new data point *p* arrives, it identifies the data membership. It scans the points located in the grids around *p* and finds the closest boundary point *p’* to *p*. The corresponding distance is denoted as *dmin*. The time complexity of this step is *O(m)*, where *m* is the number of points around the new point. If *dmin*<threshold, then *p* is the boundary point belonging to the cluster of *p’*. If *dmin*>threshold, then it calculates the boundary vector of *p’*. The time complexity of this step is still *O(m)*. According to the distance, *p* is either inside a cluster or is outside the cluster and put into a bucket.

Second, the BPIC method employs a DBSCAN process to cluster the data within the bucket when it is full. The time complexity of DBSCAN is *O(b*^*2*^*)*, where *b* is the bucket size which is a constant. If a new cluster is generated, then the BV-BPD is needed to extract its boundary profile. There are two steps in the BV-BPD. In the first step, it calculates the boundary vector of all points in the cluster generated by DBSCAN. This step’s time complexity is *O(b*^*2*^*)*, where *b* is the bucket size. At the second step, it utilizes a k-means algorithm to cluster all boundary vectors. This step’s time complexity is *O(tkpd)*, where *t* is the number of iterations, *k* is the number of clusters which equals 2, *p* is the number of data points (which is smaller than the bucket size *b*) and *d* is the dimension of the data point. Therefore, the worst time complexity of this step is *O(2tbd)*.

Third, the BPIC method updates the whole clustering results. It scans each boundary point in the newly detected boundary profile. Its time complexity is *O(qr)*, where *q* is the number of newly-detected boundary profiles and *r* is the number of points in it.

The overall time complexity of the BPIC method for dealing with a bucket is *O(2bm+b*^*2*^*+2tbd+qr)*. Since the values of *m*, *t*, *d*, *q* and *r* are generally much less than *b*, it could be *O(b*^*2*^*)*, where *b* is the bucket size. If *n* total data points are appended, then the time complexity of the whole BPIC process is *O*(⌈*n*/*b*⌉*b*^2^).

### Parameter analysis

**Fig 10:**
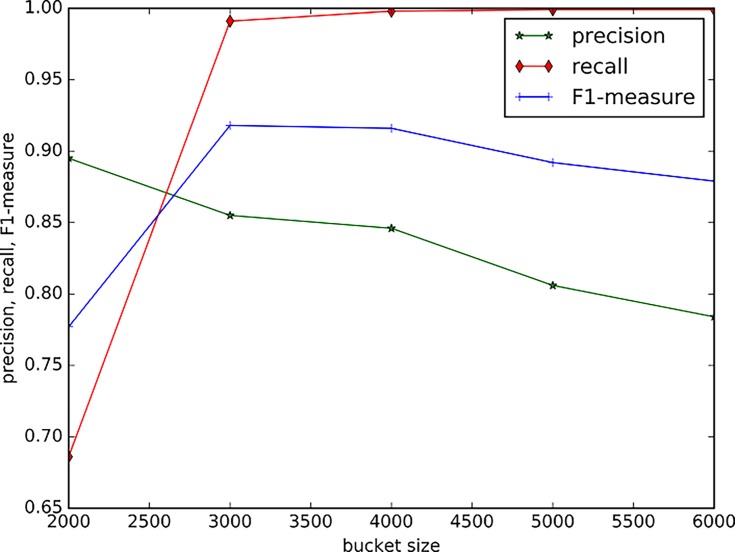
The performance of BPIC method against different size of bucket.

The bucket size is an important parameter for the BPIC method. The Bucket preserves the new uncertain data and the new boundary points. The BPIC method will update the whole clustering results when the bucket is full. If the bucket size is small, it is very likely that there will be a few seeds of new clusters within it. Thus, this small number of seeds is probably classified as noise, which is unfavourable to the emergence of new clusters. Fig 10 illustrates the performance of the BPIC method with different sizes of buckets when 90,100 data points arrive in a random order. It is shown that while the bucket size increases, the precision decreases and the recall increases.

## Conclusions

It is essential to incrementally cluster growing Big Data in many applications. This paper proposes a BPIC method that maintains the knowledge of clusters with small storage. In addition, a boundary profile provides a time efficient way to identify new data and update the clustering results. The BPIC method is tested on a large dataset from the perspective of clustering quality, time and space efficiency. The experimental results imply that the proposed BPIC method is valid and time efficient for dealing with growing Big Data.

The BPIC method has the following contributions. (1) It exploits boundary profiles to represent knowledge and discards the inner points of clusters. Thus, it greatly saves both time and space costs. (2) As an incremental clustering method, it can address noise and find arbitrarily shaped clusters. (3) It is insensitive to the order of newly added data points, which is critical for the robustness of the incremental clustering process.

At present, the BPIC method does not adopt any parallel or distributed processing strategy. In the future work, some parallel computing approaches will be explored to speed up it. In fact, many operations in the BPIC method have no sequentially dependent relationship, such as the operation of identifying new points and the operation of calculating the boundary vector of all points. So there is a huge room to further improve the performance of the BPIC method.
